# The effects of the metabolic syndrome on coronary artery bypass grafting surgery

**DOI:** 10.5830/CVJA-2016-056

**Published:** 2017

**Authors:** Sevil Özkan, Refik Demirtunç, Fatih Özdemir, Oğuz Uğur, Ahmet Yavuz Balcı, Mehmet Kızılay, Mehmet Kaplan, İbrahim Yekeler

**Affiliations:** Department of Internal Medicine, Haydarpasa Numune Training and Research Hospital, Istanbul, Turkey; Department of Internal Medicine, Haydarpasa Numune Training and Research Hospital, Istanbul, Turkey; Department of Cardiovascular Surgery, Dr Siyami Ersek Training and Research Hospital on Thoracic and Cardiovascular Surgery, Istanbul, Turkey; Department of Cardiovascular Surgery, Dr Siyami Ersek Training and Research Hospital on Thoracic and Cardiovascular Surgery, Istanbul, Turkey; Department of Cardiovascular Surgery, Dr Siyami Ersek Training and Research Hospital on Thoracic and Cardiovascular Surgery, Istanbul, Turkey; Department of Cardiovascular Surgery, Dr Siyami Ersek Training and Research Hospital on Thoracic and Cardiovascular Surgery, Istanbul, Turkey; Department of Cardiovascular Surgery, Dr Siyami Ersek Training and Research Hospital on Thoracic and Cardiovascular Surgery, Istanbul, Turkey; Department of Cardiovascular Surgery, Dr Siyami Ersek Training and Research Hospital on Thoracic and Cardiovascular Surgery, Istanbul, Turkey

**Keywords:** coronary artery bypass grafting surgery, metabolic syndrome, postoperative morbidity and mortality

## Abstract

**Background:**

The metabolic syndrome (MS) is a clustering of factors that are associated with increased cardiovascular risk. A low-grade inflammatory process acts as the underlying pathophysiology, which suggests that the MS may have a detrimental effect on coronary interventions, including coronary artery bypass grafting (CABG) surgery performed with cardiopulmonary bypass (CPB). We aimed to evaluate the effect of the MS on morbidity and mortality rates in the early postoperative period in patients undergoing CABG.

**Methods:**

We prospectively included 152 patients (109 males and 43 females; mean age 60.1 ± 8.6 years) who underwent elective CABG on CPB between January and September 2011. Early postoperative morbidity and mortality rates were compared between subjects with and without the MS. Diagnosis of the MS was based on the American National Cholesterol Education Program Adult Treatment Panel III (NCEP ATP III) criteria.

**Results:**

Of the study group, 64 patients (42%) had the MS. The two groups were similar in age and gender. In the postoperative period, rates of atrial fibrillation, wound infection, pulmonary complications, and lengths of intubation, hospitalisation and intensive care unit stay were significantly higher in MS patients (p < 0.01). The MS was significantly associated with wound infection (OR 6.64, 95% CI: 1.72–25.75), pulmonary complications (OR 6.44, 95% CI: 1.58–26.33), arrhythmia (OR 5.47, 95% CI: 1.50–19.97) and prolonged intubation (OR 1.17, 95% CI: 1.05–1.32). The mortality rate was 3.1% in the MS group and 1.1% in the non-MS group, with no significant difference (p > 0.05).

**Conclusion:**

The MS was associated with a higher rate of early postoperative morbidity following CABG, without having a significant effect on the mortality rate.

## Background

The metabolic syndrome (MS) is a complex metabolic disturbance characterised by insulin resistance, central obesity, hypertriglyceridaemia, reduced high-density lipoprotein cholesterol, hypertension and glucose intolerance.^1^ The unifying mechanism responsible for the cluster of cardiovascular risk factors in the MS is insulin resistance, which is also a hallmark of the MS.^2^ It has been proposed that insulin resistance plays a major unifying role in increased ischaemic events in MS patients, but this mechanism and ensuing processes need clarification.^3^-^5^ As described by the the American National Cholesterol Education Program Adult Treatment Panel III (NCEP ATP III), at least three of five criteria (Table 1) have to be met for a MS diagnosis.^6^

**Table 1.** Metabolic syndrome diagnostic criteria (NCEP ATP -III)

*Metabolic syndrome diagnostic criteria*
1. Abdominal obesity (waist circumference)
• Male > 102 cm • Female > 88 cm
2. Triglycerides > 150 mg/dl (1.7 mmol/l)
3. High-density lipoprotein cholesterol
• Male < 40 mg/dl (1.04 mmol/l) • Female < 50 mg/dl (1.3 mmol/l)
4. Blood pressure 130/85 mmHg
5. Fasting blood glucose > 110 mg/dl (6.11 mmol/l)

American National Cholesterol Education Program Adult Treatment Panel (NCEP ATP III) diagnostic criteria for the metabolic syndrome.

Prevalence of the MS has been reported as approximately 35–40% in industrialised countries.7 It is an inflammatory state characterised by increased levels of adipocytokines such as tumour necrosis factor-α, interleukin-6 and C-reactive protein, as well as free fatty acids, which cause vasoconstriction and endothelial dysfunction. The MS is also described as a low-grade inflammatory state manifested by increased circulating levels of inflammatory cytokines. Reduced plasma adiponectin and elevated leptin and resistin levels have been observed in MS patients. However, unlike leptin and resistin, which stimulate the immune system, adiponectin inhibits the inflammatory process in the vascular wall, mainly by inhibiting the nuclear factor kappa B pathway.^2^ The pro-inflammatory state associated with the MS may play a contributory role in exacerbation of the systemic inflammatory response induced by cardiopulmonary bypass (CPB) and surgical trauma, and therefore may predispose patients to peri-operative complications.^8^

The MS is a cluster of metabolic perturbations largely resulting from abdominal obesity, which is associated with increased risk for type 2 diabetes and cardiovascular disease.^9^ Although it has been shown to be a predictor of adverse events after cardiovascular interventions, and its association with early and late mortality and morbidity following coronary artery bypass graft (CABG) surgery has recently been reported,^10^ severalstudies failed to find such an association.^11^-^13^ We hypothesised that the MS could adversely affect the outcome in patients undergoing CABG surgery and designed a prospective study to determine the impact of the MS on postoperative morbidity and mortality rates after CABG.

## Methods

We prospectively enrolled 152 consecutive patients who underwent elective CABG at Siyami Ersek Thoracic and Cardiovascular Surgery Centre, Istanbul, Turkey, between January and September 2011. Diagnosis of the MS was made according to the NCEP ATP III criteria. Patients were divided into two groups (with and without the MS) depending on the MS diagnosis.

Pre-operative and operative data of all patients were prospectively collected and transfered to a computerised database. Demographic features, and clinical, laboratory and intensive care unit (ICU) data of the patients were obtained by trained personnel supervised by a nurse author, as well as data on risk factors, medications and functional status. Postoperative complications were recorded prospectively by an author, and all major adverse events were simultaneously validated by an experienced cardiac surgeon according to standardised definitions.

Patients undergoing emergency surgery, re-operative surgery, CABG on a beating heart, additional valve repair or replacement, having an ejection fraction of less than 45%, requiring pre-operative pacemaker implantation, and those with liver failure were excluded from the study. The study protocol was approved by the institutional review board of the hospital.

Demographic and clinical features included age, gender, mean blood pressure, body mass index (BMI), waist circumference, smoking status and co-morbidities, including type 2 diabetes mellitus, systemic hypertension and obesity. Weight was measured in kilograms using a calibrated digital scale, height was measured in centimetres using a calibrated stadiometer (Seca GmbH & Co, Germany) and body mass index (BMI) was calculated. Waist circumference was measured by a trained nurse, with a cloth tape around the waist placed in a mid-axillary line at the midpoint between the highest point of the iliac crest and the lowest part of the costal margin. Diabetes mellitus was defined as the use of diabetes medications or fasting plasma glucose concentration of ≥ 110 mg/dl (6.11 mmol/l).

The patients’ characteristics included the following: age, gender, height, BMI, waist circumference, duration of diabetes, alcohol consumption, use of insulin or anti-diabetic drugs, low-density lipoprotein (LDL-C) and high-density lipoprotein cholesterol (HDL-C), triglyceride and fasting blood glucose levels, smoking status, levels of postprandial blood glucose (PPBG), blood urea nitrogen (BUN), creatinine, aspartate aminotransferase (AST), alanine aminotransferase (ALT), HbA1c, haematocrit, haemoglobin, thyroid stimulating hormone (TSH) and free T_4_, number of grafts used during CABG, left ventricular ejection fraction, and percentage of carotid artery stenosis on Doppler ultrasonography.

Blood pressure (BP) measurements were made pre-operatively using a mercury sphygmomanometer with the patient in a sitting position following at least a 10-minute rest. The average of three measurements taken at two-minute intervals was defined as clinical BP. Hypertension was defined as BP being ≥ 140/90 mmHg from at least two measurements or the use of antihypertensive therapy. A total cholesterol level of > 200 mg/ dl (5.18 mmol/l) or a history of elevated serum total cholesterol during the past six months resulting in lipid-lowering drug use was defined as hyperlipidaemia. Current smokers and former smokers who had stopped smoking within the past three years were considered smokers.

Peri-operative variables included the number of CABG surgeries, number of grafts, cardiopulmonary bypass time (min) and aortic cross-clamp time. Postoperative variables were all-cause mortality, death within one month after the operation, renal failure, postoperative creatinine level > 2.5 mg/dl (221 mmol/l), need for haemodialysis, the use of prolonged pulmonary ventilator > 24 hours, acute myocardial infarction, ST-segment changes, prolonged ventilation (more than 72 hours), re-intubation, wound infection, stroke and regional neurological dysfunctions that resolved within 24 hours with no sequela. Additional data included the length of ICU and hospital stay.

Under local anaesthesia, radial and pulmonary arterial catheters were introduced. In all patients, anaesthesia induction was obtained before tracheal intubation using midazolam 0.05–0.1 mg/kg, fentanyl 4–8 μg/kg or sufentanil 0.6–0.8 μg/kg, atracurium 0.5 mg/kg or pancuronium 0.1 mg/kg and thiopental sodium 1–2 mg/kg. All operations were performed under CPB at mild to moderate hypothermia (28–32°C). Myocardial protection was ensured by intermittent antegrade or combined antegrade and retrograde saline or blood cardioplegia. Operative outcomes included the CPB time and aortic cross-clamp time.

## Statistical analysis

Statistical analysis was done using the NCSS 2007 software (Number Cruncher Statistical System, LCC Statistical Software, Utah, USA). Data are expressed with descriptive statistics using mean ± standard deviation, median, frequency and percentage. The Kolmogorov–Smirnov test was used to assess the compliance of numerical variables with normal distribution. The two groups were compared with regard to pre-operative demographic data, operative data and early postoperative morbidity and mortality rates. The Student’s t-test was used for intergroup comparisons of normally distributed variables, including age, BMI, female and male waist circumference, ejection fraction, number of grafts, CPB, aortic cross-clamp time, PPBG, BUN, creatinine, total cholesterol, LDL-C, HDL-C, haematocrit, haemoglobin, free T_4_ and HbA_1c_ values.

Variables that did not show a normal distrubution (EuroSCORE, fasting blood glucose, AST, ALT, triglycerides, TSH, drainage, ICU stay, hospital stay, erethrocyte sedimentation rate and fresh frozen plasma) were compared using the Mann– Whitney U-test. For the comparison of categorical variables, Pearson’s chi-squared test was used when expected and observed counts were sufficient, Yates’ correction for continuity test was used when observed counts were insufficient (< 20), and Fisher’s exact test was used when expected counts were insufficient (< 5). A p-value < 0.05 was considered statistically significant. A post hoc power analysis showed the adequacy of the sample size for further analyses.

## Results

There were 109 males and 43 females with a mean age of 60.1 ± 8.6 years (range 39–85 years). According to the NCEP ATP III criteria,3 64 patients (42%) had the MS, while the remaining 88 (58%) were free of the MS. The two groups were similar with regard to age and gender. All the MS parameters (BMI, waist circumference, rates of hyperlipidaemia, hypertension and diabetes) were significantly higher in the MS group.

Pre-operative demographic features and operative data for each study group are shown in Table 2. When compared with patients without the MS, those with the MS had higher levels of fasting glycaemia, postprandial glycaemia, plasma total cholesterol, triglycerides and LDL-C, and a lower HDL-C concentration. Laboratory findings of the two patient groups are shown in Table 3. Overall, 102 patients (67.2%) had diabetes. Medications of the diabetic patients included oral anti-diabetic agents (58.8%), insulin (18.6%) or both (10.8%), while 11.8% had been receiving no diabetes treatment.

**Table 2.** Comparison of pre-operative demographic and peri-operative data of patients with and without the metabolic syndrome

**	*MS (+) (n = 64)*	*MS (–) (n = 88)*	*p-value*
Age (mean ± SD)	59.98 ± 6.89	60.55 ± 9.74	^a^0.678
Gender, n (%)			
Male	44 (68.8)	65 (73.9)	^c^0,611
Female	20 (31.3)	23 (26.1)	
BMI (kg/m^2^) (mean ± SD)	31.09 ± 5.56	27.58 ± 3.34	^a^0.001**
Waist circumference (mean ± SD)			
Total	106.92 ± 10.30	93.69 ± 8.21	^a^0.001**
Female	108.86 ± 9.18	95.11 ± 7.77	^a^0.001**
Male	102.65 ± 11.54	89.65 ± 8.25	^a^0.001**
Smoking, n (%)	32 (50.0)	40 (45.5)	^d^0.579
Alcohol consumption, n (%)	11 (17.2)	15 (17.0)	^c^1.000
Hyperlipidaemia, n (%)	41 (64.1)	31 (35.2)	^c^0.001**
Hypertension, n (%)	57 (89.1)	11 (12.5)	^c^0.001**
Carotid Doppler USG (50 and 70%), n (%)	16 (25.0)	11 (12.5)	^d^0.076
EF% (mean ± SD)	51.25 ± 9.21	52.52 ± 10.67	^a^0.433
EuroSCORE (min–max/median)	0–9/4	0–11/4	^b^0.391
Number of grafts (mean ± SD)	3.05 ± 0.93	3.07 ± 0.84	^a^0.883
CPB (min) (mean ± SD)	74.09 ± 16.70	71.90 ± 19.82	^a^0.174
Aortic cross-clamp time (min) (mean ± SD)	51.08 ± 14.76	47.28 ± 17.84	^a^0.225

aStudent’s t-test; bMann–Whitney U-Test; cYates continuity correction test; dPearson’s chi-squared test *p < 0.05 **p < 0.01.

BMI: body mass index, EF: ejection fraction, CPB: cardiopulmonary bypass (presence of the MS was significantly associated with higher prevalence).

**Table 3:** Comparison of laboratory findings of the patients with and without the metabolic syndrome

**	*MS (+) (n = 64)*	*MS (–) (n = 88)*	*p-value*
FBG (mg/dl) (min–max/median)	79–300/151.00	77–300/110.00	^b^0.001**
PPBG (mg/dl) (mean ± SD)	191.84 ± 51.09	157.06 ± 53.04	^a^0.001**
BUN (mg/dl) (mean ± SD)	18.25 ± 5.93	17.53 ± 5.66	^a^0.447
Creatinine (mg/dl)(mean ± SD)	0.95 ± 0.26	0.96 ± 0.25	^a^0.775
AST (U/l) (min–max/median)	12–62/25.00	11–51/24.00	^b^0.490
ALT (U/l) (min–max/median)	10–97/28.00	8–61/22.00	^b^0.241
Total cholesterol (mg/dl)	226.95 ± 34.18	185.69 ± 36.08	^a^0.001**
(mean ± SD) (mmol/l)	(2.56 ± 0.89)	(4.81 ± 0.93)	
LDL-C (mg/dl) (mean ± SD)	134.92 ± 21.80	121.89 ± 25.04	^a^0.001**
^a^(mmol/l)	(3.49 ± 0.56)	(3.16 ± 0.65)	
(mmol/l)	(0.94 ± 0.24)	(1.00 ± 0.21)	
HDL-C (mg/dl) (mean ± SD)	36.22 ± 9.21	38.77 ± 7.95	^a^0.069
Triglycerides (mg/dl) (min–max/median) (mmol/l)	71–465/160.00 (0.8–5.25/1.81)	57–482/138.00 (0.64–5.45/1.56)	^b^0.049*
Haematocrit (%) (mean ± SD)	39.90 ± 4.35	41.13 ± 4.57	^a^0.098
Haemoglobin (g/dl) (mean ± SD)	13.32 ± 1.62	14.01 ± 1.60	^a^0.010*
TSH (µIU/ml) (min–max/median)	0.01–11.34/1.45	0.12–19.00/1.41	^b^0.621
Free T4 (ng/dl) (mean ± SD)	1.26 ± 0.22	1.20 ± 0.19	^a^0.071
HbA1c (%)	7.86 ± 1.59	6.61 ± 1.31	^a^0.001**

aStudent’s t-test; bMann–Whitney U-test *p < 0.05 **p < 0.01.

FBG: fasting blood glucose, PPBG: postprandial blood glucose, BUN: blood urea nitrogen, ALT: alanine aminotransferase, AST: aspartate aminotransferase, LDL-C: low-density lipoprotein cholesterol, HDL-C: high-density lipoprotein cholesterol, TSH: thyroid-stimulating hormone, HbA1c: haemoglobin A1c.

Postoperative clinical outcomes are shown in Table 4. Postoperative mortality rates were similar in the two groups, being 3.1% (n = 2) and 1.1% (n = 1) in patients with and without the MS, respectively. However, manifestations of postoperative morbidity differed significantly, with higher rates of atrial fibrillation (AF), wound infection, pulmonary complications, prolonged intubation, and longer durations of ICU stay and hospitalisation in patients with the MS (p < 0.01). The other periand postoperative findings (postoperative revisions, incidences of renal impairment, stroke, drainage, need for erythrocytes, fresh frozen plasma replacements, cardiopulmonary bypass time and aortic cross-clamp time) were similar between the two groups.

**Table 4:** Comparison of postoperative results of the patients with and without the metabolic syndrome

**	*MS (+) (n = 64)*	*MS (–) (n = 88)*	*p-value*
Prolonged intubation time (h) (min–max/median)	6–20/11.00	4–28/9.00	^b^0.001**
Drainage (ml) (min–max/median)	400–2500/650.00	200–1500/ 650.00	^b^0.135
Length of ICU stay (h) (min–max/median)	17–168/24.00	6–96/22.00	^b^0.003**
Length of hospital stay (days) (min–max/median)	4–35/7.00	1–35/7.00	^b^0.001**
RBC replacement (units/patient) (min–max/median)	0–5/2.00	0–5/2.00	^b^0.121
FFP replacement (units/patient) (min–max/median)	0–6/2.00	0–12/2.0	^b^0.153
Mortality, n (%)	2 (3.1)	1 (1.1)	^e^0.573
Myocardial infarction, n (%)	4 (6.3)	1 (1.1)	^e^0.162
Surgical revision, n (%)	5 (7.8)	5 (5.7)	^e^0.743
Renal failure, n (%)	4 (6.3)	1 (1.1)	^e^0.162
Wound infection, n (%)	14 (21.9)	3 (3.4)	^c^0.001**
Pulmonary complications, n (%)	11 (17.2)	3 (3.4)	^c^0.009**
Stroke, n (%)	4 (6.3)	1 (1.1)	^e^0.162
Atrial fibrillation, n (%)	13 (20.3)	4 (4.5)	^c^0.005**

^b^Mann–Whitney U-Test; cYates continuity correction test; eFisher’s exact test; *p < 0.05; **p < 0.01.

RBC: red blood cells, FFP: fresh frozen plasma.

A statistically significant relationship was found between the MS and wound infection (OR 6.64, 95% CI: 1.72–25.75), pulmonary complications (OR 6.44, 95% CI: 1.58–26.33), AF (OR 5.47, 95% CI: 1.50–19.97) and prolonged intubation (OR 1.17, 95% CI: 1.05–1.32). In post hoc power analysis, the observed power for wound infection, pulmonary complications, AF and prolonged intubation time were 0.944, 0.804, 0.843 and 0.715, respectively.

## Discussion

As MS patients have a high risk of developing coronary artery disease, they should be evaluated in line with coronary artery disease guidelines.^10^-^14^,^15^The NCEP ATP III stressed the cardiovascular risk factors associated with the MS.^6^ In Turkey, prevalence of the MS is as high as three out of every eight people in the adult population.^16^ Among coronary artery disease patients, its prevalence is 42.7% in males and 64.0% in females, with an overall prevalence of 53.0%.^16^ In our study, 42% of the patients had the MS, which is similar to other studies in MS patients undergoing CABG (42, 48, 47%).^11^,^12^,^17^

Although not statistically significant, MS patients also had a higher smoking rate, which reflected their habits and lifestyle. Patients with and without the MS did not differ in mortality rates; mortality occurred in two patients in the MS group (3.1%) and in one patient in the non-MS group (1.1%).

Other studies reported similar mortality rates in the two patient groups.^11^,[Bibr R12][Bibr R17] Swart et al.11 compared 370 patients with the MS (as defined by the International Diabetes Federation and NCEP ATP III criteria) and 503 patients without the MS in terms of mortality and morbidity rates following CABG. The two groups had a similar age distribution and had mortality rates of 1.9 and 1.6%, respectively (p = 0.7348). The average EuroSCORE differed significantly between the two groups, being 3.26 (median 3) in the MS group and 3.61 (median 3) in the non-MS group (p = 0.0494). The rates of re-exploration, stroke, renal insufficiency, prolonged mechanical ventilation, and the need for rewiring of sternal dehiscence were similar in the two groups. The amount of mediastinal drainage was also similar (624 vs 670 ml). The need for homologous blood transfusion was less (p = 0.0012), but hospital stay was longer (p < 0.00001) in the MS group. The authors concluded that MS did not have any detrimental clinical effects on either pre-operative risk factors or outcomes after CABG.

Özyazıcıoğlu et al.^12^ examined the effects of the MS on postoperative mortality and morbidity rates in patients undergoing CABG. Compared with patients without the MS, those with the MS (NCEP ATP III criteria) had a higher incidence of wound infection (p < 0.05), but similar rates of atrial fibrillation, revision surgery due to haemorrhage, ventricular tachycardia, ventricular fibrillation, prolonged intubation and mortality rates.

These discrepancies may have resulted from differences in the definition of postoperative morbidity and postoperative serious events, and in the duration of follow-up periods. Criteria used to define the MS may also lead to discrepant results, namely, cut-off points of criteria for the MS in various populations or even parameters of the MS (waist circumference instead of BMI) may vary. These differences may have a confounding effect on assessing the association between pre-operative MS and postoperative complications.^11^,^12^

Inhibition of adipocytes is increased in obese people, along with many proteins with immunomodulatory activity. Thromboembolic events are more commonly seen in MS patients undergoing CABG because a prothrombotic state frequently occurs postoperatively.^2^ Yılmaz et al.^18^ suggested that the MS might serve as a predictor of postoperative occlusion of saphenous vein grafts after CABG. In our study, the incidences of peri-operative myocardial infarction were similar between patients with and without the MS. It is likely that peri-operative myocardial infarction is not determined by early graft occlusion, but rather by factors related to myocardial protection strategies or unknown factors, which could explain the absence of a significant difference between patients with and without the MS.

In the present study, lengths of hospitalisation and ICU stay were significantly longer in the MS group. Brackbill et al.^13^ showed that female patients with the MS undergoing CABG surgery were at increased risk for longer postoperative stay as well as for in-hospital death. Bardakcı et al.^19^ reported that, compared with the patients without the MS, those with the MS had a significantly higher female-to-male ratio, and significantly higher rates of family history of ischaemic heart disease, and coronary artery occlusions involving the anterior descending coronary, circumflex and right coronary arteries. This difference could be noteworthy not only for increased morbidity rates, but also for treatment costs.

Similar to previous studies,^11^,^12^,^17^,^20^ no significant difference was found in the occurrences of stroke and renal impairment after CABG between the MS and non-MS groups (p < 0.05). However, many parameters of morbidity, including AF, wound infection, pulmonary complications, prolonged intubation, and lengths of ICU and hospital stay were significantly higher in patients with the MS (p < 0.01). Ardeshiri et al.^20^ found that the MS represented an increased risk for atelectasis and that patients with the MS had a longer ICU stay following CABG. Özyazıcıoğlu et al.^12^ concluded that wound infection was significantly more frequent in coronary artery disease patients with the MS than in those without the MS (p < 0.05). In a multivariate analysis, the odds ratios of postoperative stroke and renal failure in MS patients were found to be 2.47 and 3.81, respectively.^17^

The high prevalence of postoperative events in MS patients may be associated with BMI and an increased incidence of diabetes.^21^ Bardakçı et al.^19^ found significantly prolonged intubation times, ICU and hospital stay, and a significantly higher rate of pulmonary complications in MS patients; however, in contrast with our study, they reported significant increases in the rates of mortality and peri-operative myocardial infarction.

Moulton et al.^22^ reported that obesity was not a risk factor for adverse events after cardiac surgery, except for the increased number of superficial surgical wound infections and a higher incidence of atrial arrhythmias. Kopelman et al.^23^ concluded that thoracic and abdominal adipose tissue might be a cause of ventilation and perfusion mismatch, which could induce a decline in respiratory function by creating resistance to breathing exercises.

In our study, pulmonary complications were significantly higher among patients with the MS (p < 0.01). This may be explained by a negative effect of the MS on postoperative respiratory function, leading to increased postoperative pulmonary complications. Concerning the relationship between pulmonary function and the MS, it was shown that male adults with the MS had decreased vital capacity.^24^ Bagheri et al.^25^ indicated that BMI was not a predictor of mortality after CABG, but pulmonary complications were independent predictors of mortality in the postoperative period.

Cardiopulmonary bypass procedures are related to inflammatory response and free radical accumulation.^8^ It is known that MS patients have an ongoing, low-grade inflammatory process, which can be exacerbated during surgery. They also have increased systemic oxidative stress caused by oxidative transformation of LDL-C.^26^ The role of lipolytic activity by abdominal fat storage has been emphasised in the production of free fatty acids.^26^ These free fatty acids exert a significant pro-arrhythmic effect in ischaemic events. This effect has been documented for ventricular arrhythmogenicity, but it has yet to be demonstrated in the generation of AF. Therefore, further research is needed to clarify whether, like other factors, free fatty acid burden associated with hyperlipolytic visceral fat storage contributes to the generation of postoperative AF.^27^

It is believed that MS patients are more prone to postoperative AF through a potential pathway.^28^ Atrial remodelling involves two substrates: atrial architecture acting as an anatomical substrate (involved in atrial dilatation, fibrosis), and electrical inhomogeneity acting as a functional substrate (involved in shortness of effective refractory period, dispersion of refractoriness and conduction, abnormal automaticity, and anisotropic conduction).^29^ These latter processes have been shown to be potential substrates for postoperative AF.^30^

Bell and O’Keefe reported that postoperative AF was observed in 25% of patients undergoing CABG, and was associated with elevated rates of mortality and postoperative stroke, prolonged hospital stay and increased cost of hospitalisation.^31^ Kara et al.^32^ found that the incidence of AF was high (19.2%) after CABG, and they defined some independent clinical predictors.

Echahidi et al.^2^ reported that the MS had a significant effect on clinical outcomes after cardiac surgery and was an independent predictor of postoperative AF. Girerd et al.33 showed a significant correlation between postoperative AF and increased waist circumference and/or increased C-reactive protein levels. The authors also reported that the MS was an independent risk factor for AF occurring after CABG.^32^ In our study, the rate of AF was significantly higher (20.9%) in MS patients compared to those without the MS (p < 0.01).

Gharipour et al.^34^ found no significant difference in the incidence of postoperative stroke between CABG patients with and without the MS. In our study, although the rate of stroke was higher in MS patients (6.3 vs 1.1%), it was not associated with a significant difference (p = 0.162). This may be attributed to the absence of atherosclerotic plaque in the carotid arteries.

In our patients, carotid Doppler ultrasound showed moderate stenosis (50–70%), which was considered insufficient to lead to haemodynamically significant conditions. Carotid stenosis is an important risk factor for stroke during CABG surgery, but neurological events may develop from other causes as well, including aortic and carotid atherosclerosis (62%), intracardiac thrombi (1%), haemorrhage (1%), hypoperfusion (11%), and other factors of unknown origin (25%).35 The severity of carotid stenoses detected in patients undergoing CABG has been reported as greater than 70% in 10% of the cases, 50–70% in 9–22% of the cases, and less than 50% in 80–91% of cases.^35^,^36^ Of interest, 50–75% of patients who suffered a stroke did not have carotid stenosis.^37^ Lee et al.^38^ reported that intracranial atherosclerosis was the main determinant of stroke, while extracranial atherosclerotic processes played a relatively smaller role.

Of note, pre-operative critical risk factors for mortality after CABG were not affected by the MS. By contrast, patients without the MS required urgent operations more frequently than did those with the MS. This is not surprising because patients with the MS are normally on strict follow up to control hypertension, diabetes mellitus and dyslipidaemia, all of which are known to be underlying risk factors for coronary artery disease. Therefore, patients without the MS and with poorly controlled coronary risk factors are more likely to have urgent, non-elective interventions.

The presence of factors known to increase mortality rates in MS patients may itself be a limitation to the study. These factors include male gender, widespread coronary artery involvement, and increased cross-clamping time. Therefore, the effect of the MS on mortality rate itself may be considered a limitation. The biggest limitation was that the study was underpowered to draw conclusions on some of the outcomes, for example, mortality.

Components of the MS cannot be completely minimised by conventional pharmacological treatment modalities. It is well known that statins, angiotensin converting enzyme inhibitors, and beta-blockers have little or no effect in metabolic disturbances observed in MS cases.^28^

## Conclusion

Since MS patients already present with many cardiovascular risk factors, the MS was associated with increased morbidity rates in the early postoperative period after CABG; however, its effect on early mortality rate was similar to that seen in patients without the MS. Considering the increased postoperative morbidity rate, the MS should be taken into consideration in pre-operative assessment of CABG patients.
